# Supplementary material for: Parkinsonism Reversal and Dopaminergic Resilience: Lessons from a Rotenone-induced Parkinson’s Disease Model
Source: bioRxiv. 2025 Dec 23:2025.12.20.695709. Preprint. [Version 1] doi: 10.64898/2025.12.20.695709 (PMC12776045; doi:10.64898/2025.12.20.695709)
Supplement: Supplement 1 [file NIHPP2025.12.20.695709v1-supplement-1.pdf]

bioRxiv preprint doi: https://doi.org/10.64898/2025.12.20.695709; this version posted December 23, 2025. The copyright holder for this preprint (which was not certified by peer review) is the author/funder, who has granted bioRxiv a license to display the preprint in perpetuity. It is made available under aCC-BY 4.0 International license.

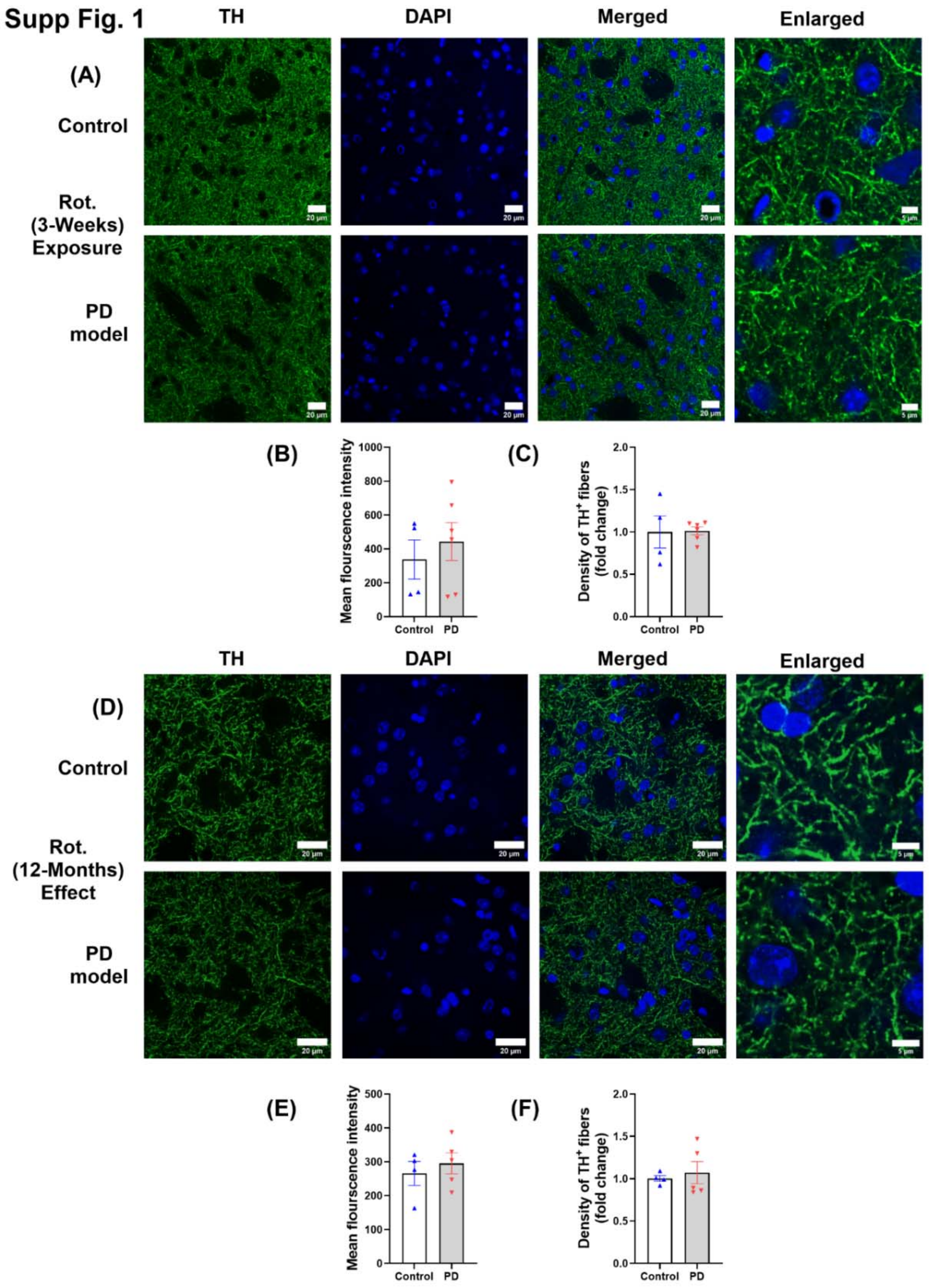

Supplementary Figure 1. Striatal TH was preserved after both short and long-term rotenone exposure in mice.

(A) Representative confocal images of TH immunofluorescence (green) and nuclear counterstain (DAPI, blue) in the striatum after 3 weeks of rotenone exposure. The far-right panel shows enlarged views of individual TH^+ axons. (B-C) Intact TH fluorescence and density in the striatal dorsal lateral region at 1 month. (D-F) like (A-C) but at 12 months. Data are presented as mean ± SEM, Mann-Whitney nonparametric test**;** 3-weeks (control n = 4, PD model n = 6), 12-months (control n = 4, PD model n = 5).

bioRxiv preprint doi: https://doi.org/10.64898/2025.12.20.695709; this version posted December 23, 2025. The copyright holder for this preprint (which was not certified by peer review) is the author/funder, who has granted bioRxiv a license to display the preprint in perpetuity. It is made available under aCC-BY 4.0 International license.

Supp Fig. 2

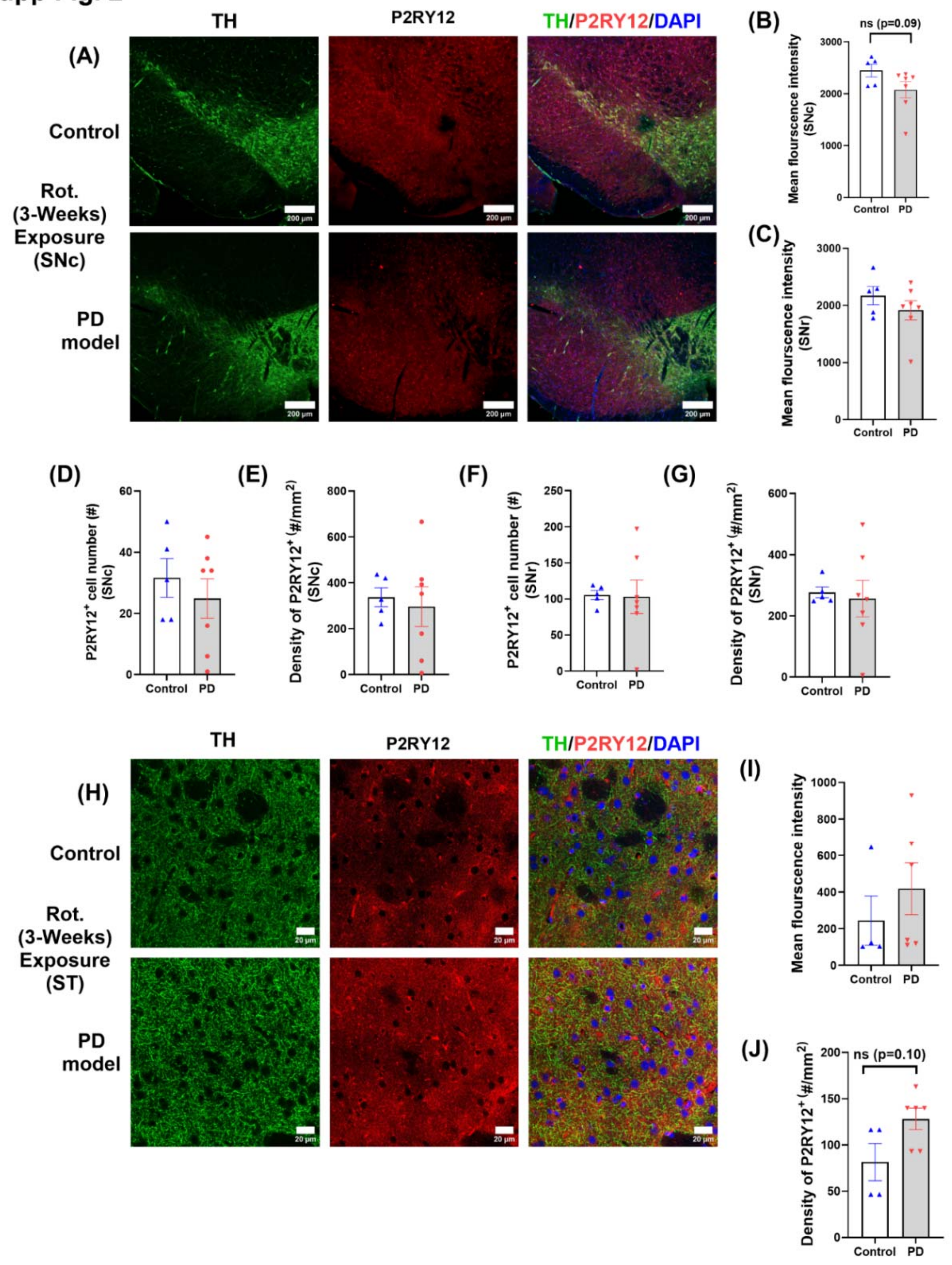

Supplementary Figure 2. Characterization of microglial P2RY12 changes in SNc and striatum at the acute phase of rotenone exposure.

(A) Representative confocal images of SNc showing TH (green), P2RY12 (red), and nuclei (DAPI; blue) from coronal sections of control and rotenone-treated mice after 3 weeks of rotenone treatment. (B-C) Microglial P2RY12 fluorescence intensity in the SNc and SNr. (D-E) P2RY12□ microglial cell number and cell density in the SNc, and (F-G) P2RY12□ microglial cell number and cell density in the SNr. (H) Confocal images of TH (green), P2RY12 (red), and nuclei (DAPI; blue) in the dorsal lateral striatum. (I-J) Intact P2RY12 fluorescence intensity and P2RY12□ cell density. Data are presented as mean ± SEM; Mann-Whitney nonparametric tests; SNC (control n = 5, PD model n = 7), striatum (control n = 4, PD model n = 6); ns, not significant.

bioRxiv preprint doi: https://doi.org/10.64898/2025.12.20.695709; this version posted December 23, 2025. The copyright holder for this preprint (which was not certified by peer review) is the author/funder, who has granted bioRxiv a license to display the preprint in perpetuity. It is made available under aCC-BY 4.0 International license.

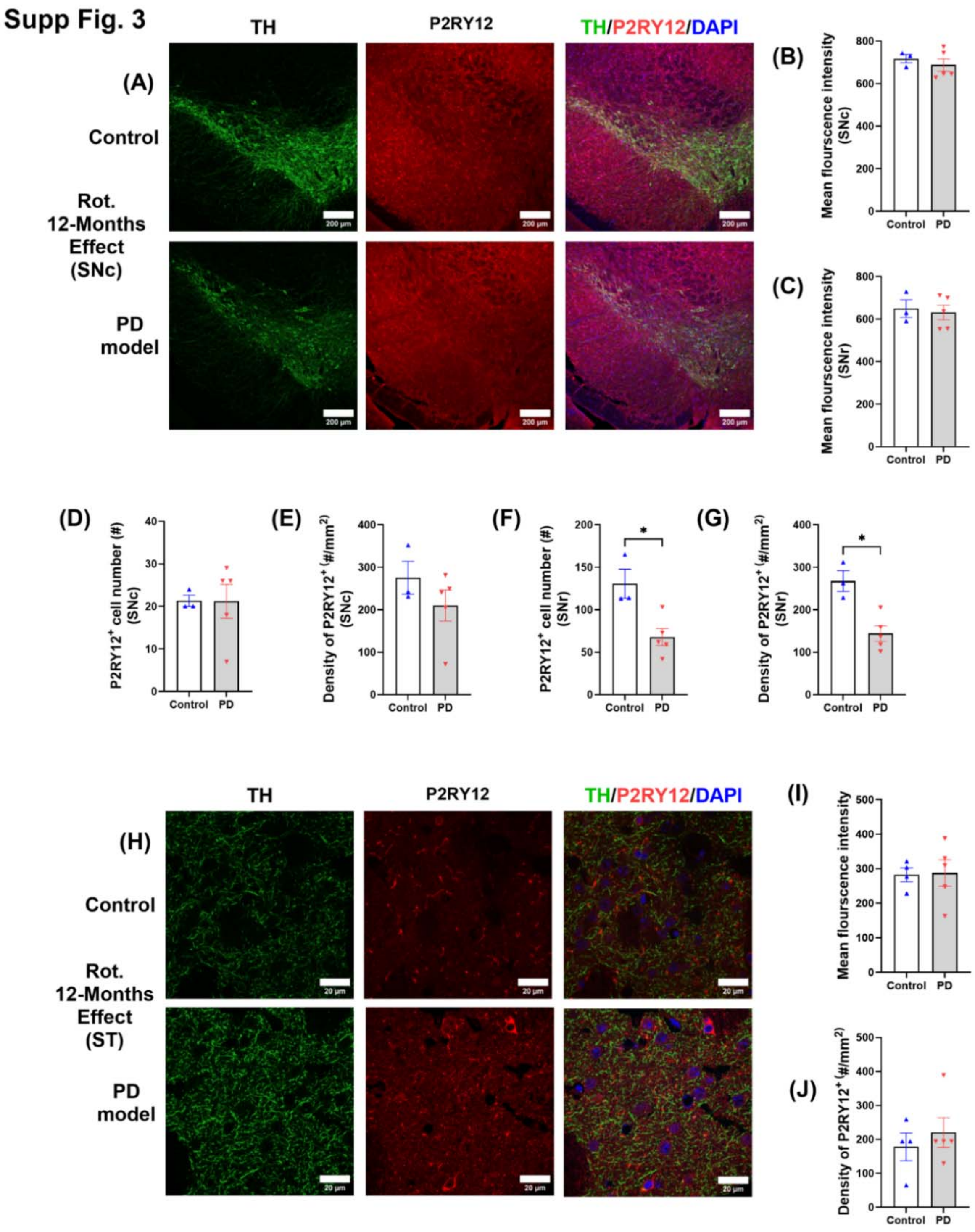

Supplementary Figure 3. Microglial P2RY12 changes in the SNc and striatum at 12 months.

(A) Representative confocal images of SNc regions showing TH (green), P2RY12 (red), and nuclei (DAPI; blue) from control and rotenone-treated mice at 12 months. (B-C) Microglial P2RY12 fluorescence intensity in the SNc and SNr. (D-E) P2RY12□ microglial cell number and cell density in the SNc. (F-G) P2RY12□ microglial cell number and cell density in the SNr. (H) Confocal images of TH (green), P2RY12 (red), and nuclei (DAPI; blue) in the dorsal lateral striatum at 12 months. (I-J) Intact P2RY12 fluorescence intensity and P2RY12□ cell density. Data are presented as mean ± SEM; two-tailed Welch's *t* test; SNc (control *n* = 3, PD model *n* = 5), striatum (control *n* = 4, PD model *n* = 5); ns, not significant.
